# A Case of Classic Hodgkin Lymphoma Involving the Uterine Cervix Presenting As Vaginal Spotting

**DOI:** 10.7759/cureus.8889

**Published:** 2020-06-28

**Authors:** Dereen Mohammed Saeed, Pritesh Patel, Grace Guzman, Hongyu Ni

**Affiliations:** 1 Pathology, University of Illinois at Chicago, Chicago, USA; 2 Hematology and Oncology, University of Illinois at Chicago, Chicago, USA; 3 Pathology and Laboratory Medicine, University of Illinois at Chicago, Chicago, USA; 4 Hematopathology, University of Illinois at Chicago, Chicago, USA

**Keywords:** classic hodgkin lymphoma, uterine cervix, vaginal spotting

## Abstract

Classic Hodgkin lymphoma (CHL) is a clonal lymphoid neoplasm derived from B cells. CHL usually involves the lymph nodes. Although cases with extranodal involvement by CHL have been reported, the involvement of the uterine cervix by CHL is an extremely uncommon phenomenon. Herein, we report an unusual case of a 51-year-old female with nodular sclerosis CHL, diagnosed initially via right inguinal lymph node biopsy. After two cycles of chemotherapy, she presented with vaginal spotting and CT scan demonstrated a uterine cervical lesion with hypermetabolic activity. Tissue biopsy sections of the uterine cervix showed cellular infiltrate consisting of large atypical cells including many lacunar cells and occasional Reed-Sternberg cells in the background of mixed reactive cells including small- to medium-sized lymphocytes, histiocytes, plasma cells, eosinophils, and neutrophils. Immunohistochemical stains show that the large atypical cells are positive for CD30, CD15, MUM-1, and weakly positive for PAX-5. In situ hybridization for Epstein-Barr virus-encoded RNA (EBER) is negative. The morphological and immunohistochemical findings were consistent with involvement by nodular sclerosis CHL. This case demonstrates a rare presentation of CHL that may pose a diagnostic problem if its existence is not considered in the differential diagnosis. Furthermore, we reviewed the literature and only found two previous publications described uterine cervix involvement by CHL. Although it is very rare, CHL involvement should be included in the differential diagnosis and an appropriate work-up should be performed to evaluate CHL involvement of cervix when patients with CHL present with signs or symptoms suggesting a cervical lesion.

## Introduction

Lymphoma is a relatively common hematological malignancy. It is generally divided into two categories, Hodgkin lymphoma (HL) and the more common form non-Hodgkin lymphoma (NHL) [1]. As the name indicates, lymphoma mostly involves lymphatic tissue, such as lymph nodes and spleen. However, patients may present with the widespread disease at the time of diagnosis [1,2]. Based on the morphology and immunophenotype, HL is divided into classic Hodgkin lymphoma (CHL) and nodular lymphocyte-predominant Hodgkin lymphoma (NLPHL) [1]. CHL accounts for 90% of all HL. It mainly involves the axial body lymph nodes rather than peripheral lymph nodes seen in NLPHL [2].

CHL is further divided into four subtypes, nodular sclerosis, mixed cellularity, lymphocyte rich, and lymphocyte depleted. Nodular sclerosis subtype (NSCHL) accounting for 23.5% of all CHL cases [3,4]. CHL mostly involves mediastinal lymph nodes and less commonly spleen, lung, bone marrow, and liver [1]. Although extranodal involvement by CHL has been reported, the involvement of the uterine cervix by CHL is extremely rare [5]. We reviewed the literature and we came across very few cases of lymphoma involving the cervix, of note, only two cases of NSCHL have been reported. The majority of cases reported were diffuse large B cell lymphoma (DLBCL) and a few cases of NLPHL [4,6]. In this report, we describe an unusual case of CHL involving the uterine cervix, demonstrates the importance of including CHL as a differential diagnosis in patients who present with signing and symptom of a cervical lesion. The abstract of this paper has been published *Archives of Pathology and Laboratory Medicine *[7].

## Case presentation

**Figure 1:**
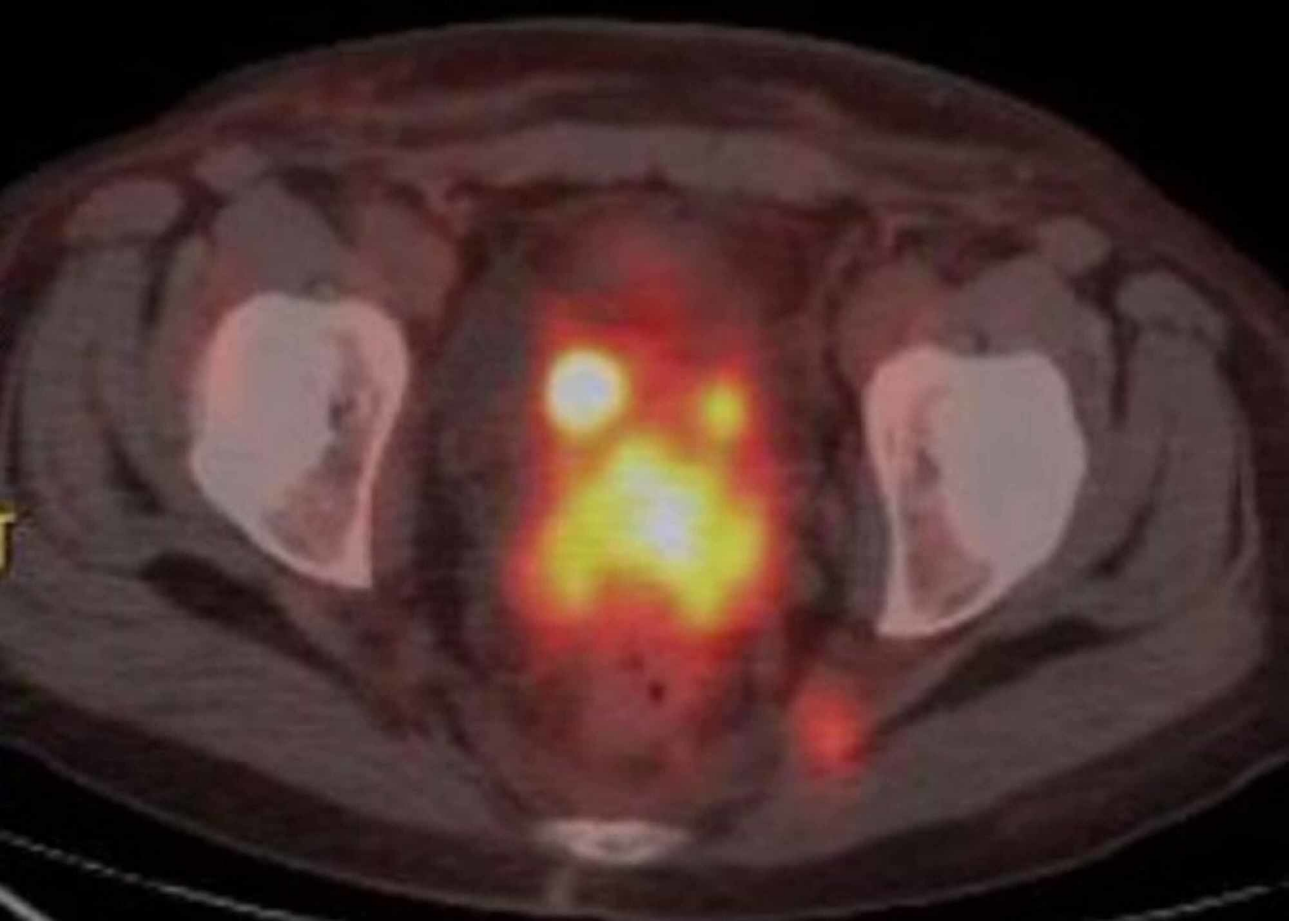
PET-CT scan of the pelvic shows focal area of marked hypermetabolic activity in the cervix PET-CT: positron emission tomography-computed tomography

The patient is a 50-year-old, multiparous, postmenopausal female with a history of CHL, nodular sclerosis type, diagnosed via right inguinal lymph node biopsy in July 2017. Biopsy revealed a few large atypical cells including Reed-Sternberg cells (R-S), which were positive for CD30, and CD15, while negative for CD3 and CD20 as demonstrated by immunohistochemical stains. Following the initial diagnosis, the patient decided to undergo holistic/natural medicine treatment including NSAIDs, Keppra, and caffeine enemas instead of conventional chemotherapy. Because of symptomatic anemia and abdominal pain, she presented to the hospital for evaluation. In January 2018, she had a hemoglobin of 6.5 g/dl, hypercalcemia, acute kidney injury, anasarca, and abnormal liver function tests. Common bile duct stenting was performed because of bile duct obstruction from enlarged lymph nodes in the right hepatic lobe. Bone marrow biopsy was performed for staging, which was negative for involvement by HL. She was given chemotherapy with ABVD regimen (adriamycin, bleomycin, vinblastine, and dacarbazine). Her PET scan in March 2018, after two cycles of ABVD, was negative for enlarged lymph node involvement and she was continued on AVD. Subsequent positron emission tomography-computed tomography (PET-CT) was done in July 2018, which showed FDG avid lymph nodes in multiple areas in the neck, thoracic cavity, abdomen, and pelvic, which were previously negative when compared to PET scan performed in March. Left inguinal lymph node biopsy was performed and confirmed the recurrence of CHL. Interestingly marked hypermetabolic activity was also noted in the uterine cervix, which was not seen in the previous imaging (Figure 1).

 

The patient was referred to the gynecology department for further evaluation. At the same time, she was also experiencing vaginal spotting for one week. Pap smear examination revealed atypical squamous cells of undetermined significance (ASCUS). Pelvic examination revealed abnormal, bulky, multinodular cervix with increased vascularity. Subsequently, a cervical biopsy was performed.

**Figure 2:**
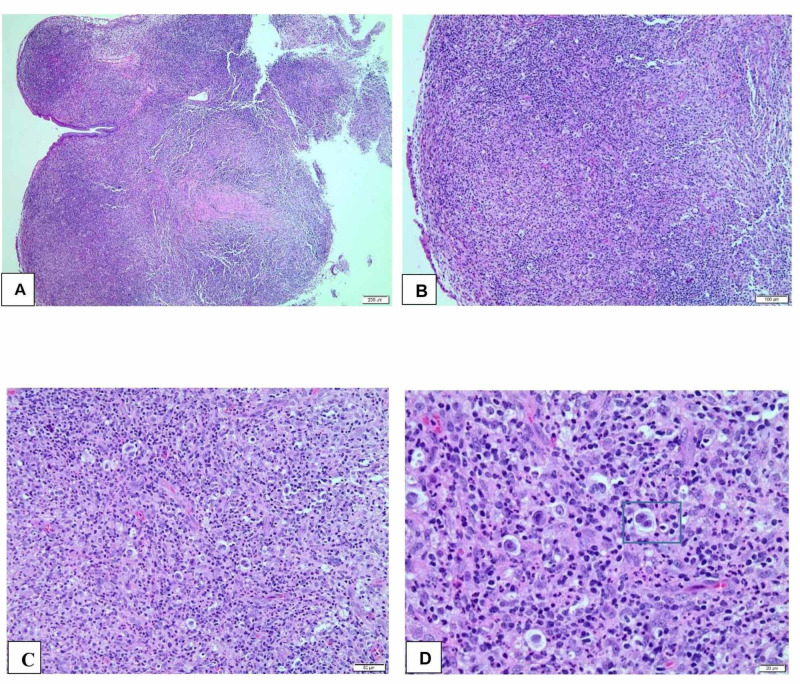
Microscopic images of the cervical lesion (A) and (B) demonstrate loss of cervical normal architecture by cellular infiltration in a nodular pattern. No cervical glands seen (H&E). (C) shows infiltration by small to medium size lymphocytes, histiocytes, plasma cells, eosinophils, and neutrophils admixed with large atypical cells including many lacunar cells (H&E). (D) Large atypical cells display amphophilic cytoplasm, multilobulated nuclei, and small eosinophilic nucleoli as indicated in the blue square (H&E) H&E: hematoxylin and eosin

Morphologic examination of cervical biopsy tissue shows cellular infiltrate consisting of small to medium-sized lymphocytes, histiocytes, plasma cells, eosinophils, and neutrophils admixed with large atypical cells including many lacunar cells (Figure 2). Some large cells exhibit binucleation and prominent nucleoli, resembling Reed-Sternberg (R-S) cells (Figure 2D).

**Figure 3:**
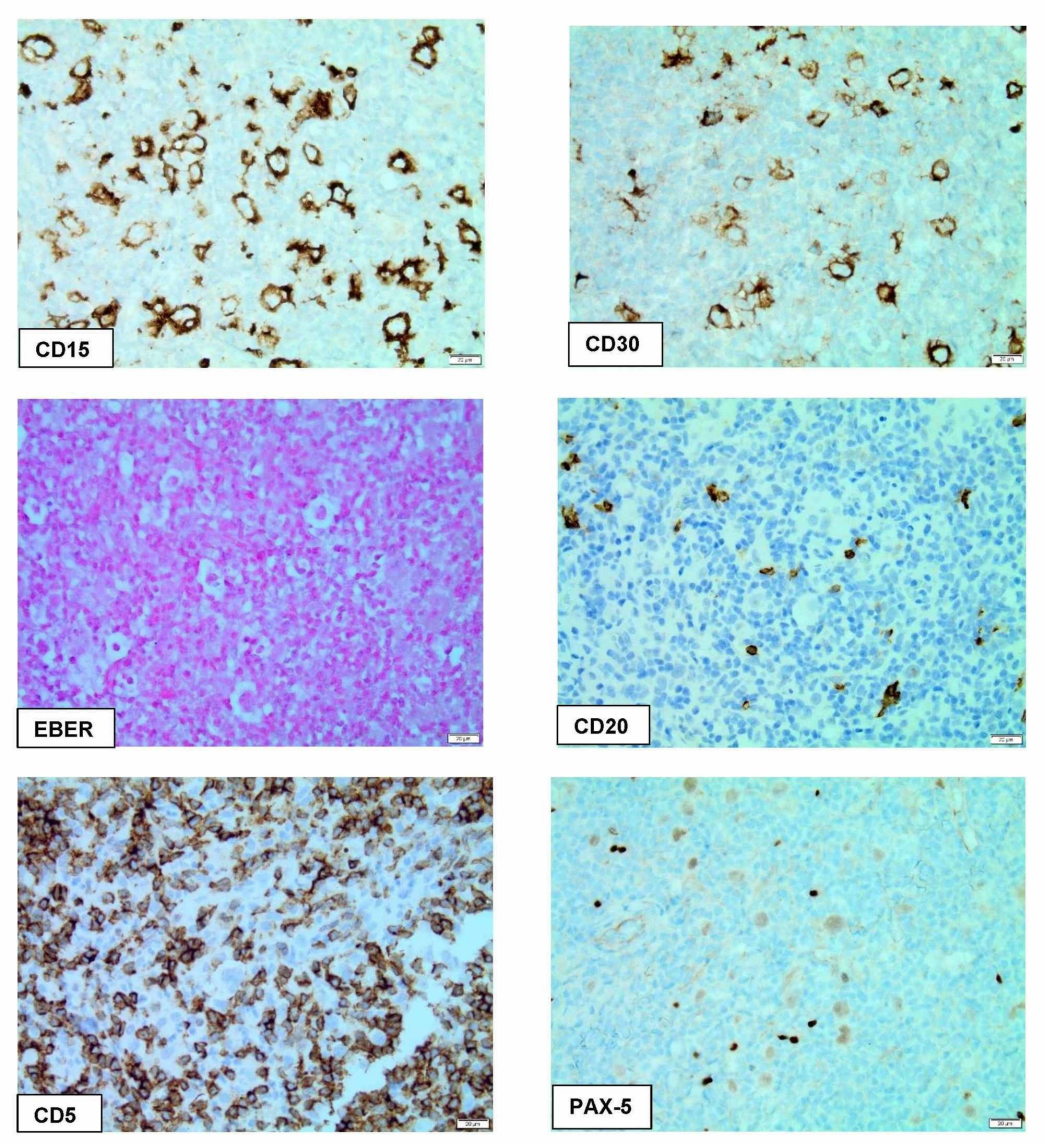
Immunohistochemical stains of the cervical lesion CD15 and CD30 are positive in large typical cells. Epstein-Barr virus-encoded RNA (EBER) in situ hybridization is negative. CD5 is positive in the background reactive T cell is negative in large atypical cells. CD20 is positive in scattered B-cells and negative in large atypical cells. PAX-5 is weakly positive in large atypical cells and strongly positive in reactive B-cells

Immunohistochemical stains show that the large atypical cells are positive for CD30, CD15, MUM-1, and weakly positive for PAX-5. In situ hybridization for Epstein-Barr virus encoded-RNA (EBER) is negative. The large atypical cells are negative for CD3, CD5, CD20, and AE1/AE3 (Figure 3). The morphological and immunohistochemical findings were diagnostic of NSCHL involving the cervix.

The patient was started on chemotherapy with ICE regimen (ifosfamide, carboplatin, etoposide) for salvage therapy. On follow-up, she was prepared for allogeneic bone marrow transplant with haploidentical stem cell transplant from her daughter and was successfully performed in January 2019. Her stem cell transplant course was complicated by GVHD treated with steroid and subsequently, she developed steroid-induced diabetes mellitus. By the end of January 2020, the patient presented with fatigue, back, pelvic, and lower extremity pain for 10 days. MRI showed multiple bone marrow signal changes and enhancement in the base of the skull and cervical spine suggestive for recurrent disease. A few days later, the patient developed diplopia, headache, and nausea. CT scan of the head was negative, but it showed a left cavernous sinus mass. CT scan of the chest showed multiple lung and liver masses as well as right side pleural effusion and right 11th rib with underlying lucency suggestive of pathological fracture. Results of liver and bone marrow biopsy, as well as pleural fluid fine-needle aspiration, were consistent with involvement by recurrent NSCHL. The patient was deceased as a consequence of multiorgan failure and septicemia.

## Discussion

Lymphoma involving the uterine cervix is very rare, and it accounts for only 1% of cervical malignancies [2,4,5]. Furthermore, uterine cervix involvement by CHL is a remarkably unusual phenomenon [4,8]. To the best of our knowledge, this is the third published case of nodular sclerosis CHL involving the uterine cervix [4,6]. Mihaljevic et al. have previously reported the first case of NSCHL of the cervix in a 69-year-old female, who was initially diagnosed with disseminated disease and treated with chemotherapy and radiotherapy. She was in remission for about 15 years and then the disease recurred with solitary involvement of the cervix [6]. In the current case, cervical involvement was part of disseminated disease rather than a solitary lesion. Therefore, herein we are reporting the second case of relapsed NSCHL involving the uterine cervix.

The most common histological type of lymphoma involving the cervix reported in the literature is DLBCL [8]. Harris and Scully have studied 25 cases of uterine lymphoma, of which most of them were diffuse large B cell lymphoma, with the remaining cases consisting of nodular lymphoma, and one case Burkitt lymphoma [9]. However, immunophenotyping was not performed in these cases to further characterize the nodular lymphoma [8,9].

HL of the cervix occurs most commonly in premenopausal women with a median age of 45 years. Vaginal bleeding or spotting is the most common presentation [9,10]. Interestingly, Raggio et al. have reported a case of HL of the uterus and cervix with signs and symptoms of pelvic inflammatory disease [10].

Cervical Pap smear is usually normal in the cases of HL with cervical involvement since the tumor located in the cervical stroma and the lining squamous epithelium is usually preserved. Therefore, it may be difficult to diagnose the HL without cervical biopsy [11]. On pelvic examination, studies have shown a diffuse enlargement of the cervix is the most common appearance for stage II-IV disease, and less frequently, polypoid or multinodular masses which may be mistaken as leiomyoma [11]. For stage I disease, the cervix is usually normal [12,13]. In our case, the cervix showed a bulky lesion which was, multinodular and hypervascular, leading to the initial clinical impression as carcinoma. Biopsy revealed diffuse cervical stromal involvement by the tumor cells with preserved surface squamous epithelium. There were abundant R-S cells with cytoplasmic artificial retraction due to fixation, which have been described as lacunar cells. 

As in other lymphoma types, immunophenotyping plays a major role in making a definitive diagnosis. The R-S cells in our case are positive for CD15, CD30, MUM-1, and weakly positive for PAX-5. The background T cells and B cells were positive for CD3 and CD20, respectively. Unlike its counterpart in NLPHL which is usually CD15 and CD30 are negative, while are positive for CD20 and PAX5. EBER was negative in our case.

Overall, the prognosis of CHL is less favorable than NLPHL with quite frequent relapses [1]. Radiotherapy is usually advised for stage I/II disease, while more advanced stages usually treated with chemotherapy and radiotherapy [12]. Therefore, the interdisciplinary cooperation involving pathologists, gynecologists, and hematologists is important to provide diagnosis and optimal treatments for these patients.

## Conclusions

The involvement of the uterine cervix by CHL should be included in the differential diagnosis of patients with a history of CHL and present with signs and symptoms suggesting a cervical lesion.
